# A systematic review of medical students’ and professionals’ attitudes and knowledge regarding medical cannabis

**DOI:** 10.1186/s42238-021-00100-1

**Published:** 2021-10-12

**Authors:** Jared M. Weisman, Marcus Rodríguez

**Affiliations:** 1 Pitzer College, Claremont, CA USA; 2 MCR Labs, LLC, Framingham, MA USA; 3 Boston Child Study Center, Los Angeles, CA USA

**Keywords:** Medical cannabis, Cannabis policy, International comparisons, Medical students, Medical professionals, Attitudes

## Abstract

**Background:**

Recently, the renewed global interest in cannabis’ therapeutic properties has resulted in shifting attitudes and legislative policies worldwide. The aim of this systematic review is to explore the existing literature on medical professionals’ and students’ attitudes and knowledge regarding medicinal cannabis (MC) to assess any relevant and significant trends.

**Methods:**

This systematic review was conducted in accordance with PRISMA guidelines. Using PubMed and Google Scholar, a literature search was performed to identify studies pertaining to healthcare professionals’ and medical students’ knowledge and attitudes regarding MC. There were no search limits on the year of publication; however, studies without primary data (e.g., abstracts, systematic reviews, meta-analyses) and non-English language papers were excluded. Studies were coded according to the following research questions: (1) Do respondents believe that cannabis should be legalized (for medicinal and/or recreational purposes)? (2) Are respondents confident in their level of knowledge regarding cannabis’ clinical applications? (3) Are respondents convinced of cannabis’ therapeutic potential? 4) What current gaps in knowledge exist, and how can the medical community become better informed about cannabis’ therapeutic uses? and (5) Are there significant differences between the knowledge and opinions of healthcare students versus healthcare professionals with respect to any of the aforementioned queries? Chi-square tests were used to assess differences between medical students and medical professionals, and Pearson’s bivariate correlations were used to analyze associations between survey responses and year of publication—as a proxy measurement to assess change over time.

**Results:**

Out of the 741 items retrieved, 40 studies published between 1971 and 2019 were included in the final analyses. In an evaluation of 21 qualified studies (8016 respondents), 49.9% of all respondents favored legalization (SD = 25.7, range: 16–97%). A correlational analysis between the percentage of survey respondents who support MC legalization and year of publication suggests that both medical students’ and professionals’ support for MC legalization has increased from 1991 to 2019 (*r*(19) = .44, *p* = .045). Moreover, medical professionals favor the legalization of MC at a significantly higher rate than students (52% vs. 42%, respectively; *χ*^2^ (1, *N* = 9019) = 50.72 *p* < .001). Also, respondents consistently report a strong desire for more education about MC and a substantial concern regarding MC’s potential to cause dependence and addiction. Pearson’s correlations between year of publication and survey responses for both of these queried variables suggest minimal changes within the last decade (2011–2019 for addiction and dependence, 2012-2019 for additional education; *r*(13) = − .10, *p* = .713 and *r*(12) = − .12, *p* = .678, respectively).

**Conclusion:**

The finding that both medical students’ and professionals’ acceptance of MC has significantly increased in recent decades—in conjunction with their consistent, strong desire for more educational material—suggests that the medical community should prioritize the development of MC educational programs. MC is far more likely to succeed as a safe and viable therapy if the medical professionals who administer it are well-trained and confident regarding its clinical effects. Limitations include a lack of covariate-based analyses and the exclusion of studies published after the literature search was performed (June 2019). Future research should analyze studies published post-2019 to draw temporal comparisons and should investigate the effect of numerous covariates (e.g., gender, religiosity, prior cannabis use) as newer studies gather data on these factors [PROSPERO Registration: CRD42020204382].

## Background

Archaeological inquiry has revealed that cannabis use has been pervasive throughout human society for at least five millennia. In fact, it was widely used as a medical therapy in the USA in the 19th and early 20th centuries and was first incorporated into the *United States Pharmacopoeia* in 1850 (Bridgeman and Abazia 2017). The first federal restrictions on cannabis occurred in 1937 with the passage of the Marihuana Tax Act, which heavily regulated its usage and sale. Subsequently, cannabis was dropped from the *United States Pharmacopoeia* in 1942, and legal penalties for its possession increased in 1951 and 1956 with the enactment of the Boggs and Narcotic Control Acts, respectively. Finally, the Controlled Substances Act of 1970 relegated cannabis to Schedule I status at the federal level, imposing limitations on research by restricting the procurement of cannabis for research purposes (Bridgeman and Abazia 2017). Moreover, cannabis remains illegal under international law. From 1961 to 2020, The United Nations’ Single Convention on Narcotic Drugs (1961) placed cannabis and its derivative products in Class IV: the most restrictive category—analogous to the DEA’s Schedule I designation (United Nations 1964). However, in December 2020, the UN Commission on Narcotic Drugs reclassified cannabis and cannabis resin to recognize its medicinal value. Reclassification will rescind some longstanding procedural barriers to research and development of cannabis-based medicinal products; however, it will not affect its recreational use or promote legalization, and it will remain under strict international control. According to international law, cannabis will now be classified as having a similar degree of abuse and dependence potential as opiate-based drugs such as morphine and oxycodone (World Health Organization 2020).

Notwithstanding, as of May 2021, 36 US states, 4 US territories, the District of Columbia, and several dozen nations around the world have passed laws permitting the renewed medicinal usage of cannabis (Bifulco and Pisanti 2015; Hanson 2021). Fortunately, dozens of studies assessing healthcare professionals’, medical students’, and patients’ knowledge and attitudes towards medicinal cannabis (MC) have been published in that time frame. Several such studies predate the first legislative bill legalizing MC in California (in 1996), and many studies were conducted between 1996 and 2019, when 32 other states and several countries—including Canada, Australia, and Ireland—legalized MC (Bridgeman and Abazia 2017; Crowley et al. 2017; Fischer et al. 2015; Hanson 2021; Thomsen 2016).

Notably, in 2019, Gardiner et al. published the first systematic review of health professionals’ beliefs, knowledge, and concerns surrounding MC (Gardiner et al. 2019). They found that healthcare providers generally supported MC use, despite a nearly unanimous lack of self-perceived knowledge regarding all of its clinical effects. Additionally, a preponderance of respondents voiced concerns about cannabis’ direct harm to patients and its indirect societal harms. While Gardiner et al.’s review provides a valuable compendium of data regarding health professionals’ general attitudes and knowledge of MC, this review broadens the scope of theirs by additionally assessing the responses of healthcare students. Furthermore, this review expands upon the following three questions investigated by Gardiner et al.: (1) How do health professionals feel about the use of MC in clinical practice? (2) How knowledgeable are health professionals regarding MC? and (3) What concerns exist for health professionals regarding the delivery of MC? With respect to question one, this review seeks to expand the scope of Gardiner et al.’s query by independently assessing respondents’ opinions regarding: medicinal versus recreational legalization; potential federal rescheduling; and clinical efficacy. With respect to question two, this review expands the scope of Gardiner et al.’s query by assessing both respondents’ self-reported knowledge regarding MC and assessing their desire for further education. Finally, with respect to question three, this review specifically assesses respondents’ concerns regarding MC’s potential to cause addiction and dependence—thereby expanding upon Gardiner et al.’s generalized query and seeking to inform future policy by addressing one of MC’s most pressing and pointed issues.

Additionally, in 2021, Zolotov et al. published a scoping review exploring the status of MC education among healthcare trainees (Zolotov et al. 2021). Their review of 23 studies across ten countries found that healthcare trainees lack sufficient knowledge about MC and do not feel prepared to counsel patients on the subject. They also found that deans and educational faculty agree on the need to educate students on MC, with an emphasis on a competencies-based curriculum. While Zolotov et al.’s review provides the most detailed investigation into MC education to date, this review expands upon their study by analyzing previously unaddressed temporal, geographic, and demographic factors. Moreover, this study provides a holistic review of MC literature, with education being one topic among several which are considered and analyzed.

Ultimately, the limited scope of Gardiner et al. and Zolotov et al.’s reviews—in addition to a preexisting wealth of published, peer-reviewed survey data addressing several other specific issues—led to the formation of the following seven guiding research questions which constituted the backbone of this novel systematic review.

1. Do you believe that physicians deserve the legal right to prescribe cannabis to patients? (i.e., Do you believe that cannabis should be legalized for therapeutic purposes?)
2. Do you believe that cannabis has any therapeutic utility?
3. Do you believe that cannabis should be legalized for recreational use?
4. [For US-based papers only] Do you believe that the USA should amend cannabis’ federal status as a Schedule 1 controlled substance (the most restrictive classification, asserting that the substance has no accepted medical use)?
5. Do you feel confident in your level of knowledge regarding the health effects of cannabis?
6. Do you desire additional education regarding MMJ and/or do believe that education on (medical) cannabis should be made readily available to medical professionals?
7. Are you concerned about cannabis’ dependence/addiction potential?

In light of the legislative hurdles and cultural stigmatization surrounding cannabis, we hope this systematic review will provide an important framework for better understanding how the medical community can work to overcome sociocultural obstacles which currently impede the acceptance of MC and other emergent, alternative therapies.

## Methods

Using both Google Scholar and PubMed, a literature search was performed between July 4th, 2019, and September 12th, 2019, to identify studies investigating healthcare students’ and professionals’ knowledge and attitudes regarding cannabis. Studies which solicited the opinions of physicians, nurses, physician’s assistants, pharmacists, and medical and pharmacy students were all deemed relevant. The searches utilized three main keyword categories: (1) keywords pertaining to various respondent types (e.g., “physician” or “health professional”); (2) keywords identifying specific types of response solicitation (e.g., “attitudes” or “opinions”); and (3) keywords corresponding to various substance-related topics (e.g., “cannabis” or “cannabinoids”). A comprehensive list of all the keywords utilized in the literature searches is provided in Appendix A. Moreover, the reference lists of selected papers were assessed to identify additional studies of relevance, and both databases provide investigators with extensive lists of related studies—helping to augment the simple keyword search protocol. The entire protocol was conducted in accordance with PRISMA guidelines and was registered and added to the University of York’s PROSPERO systematic review database, and given the ID number: CRD42020204382.

**Fig. 1:**
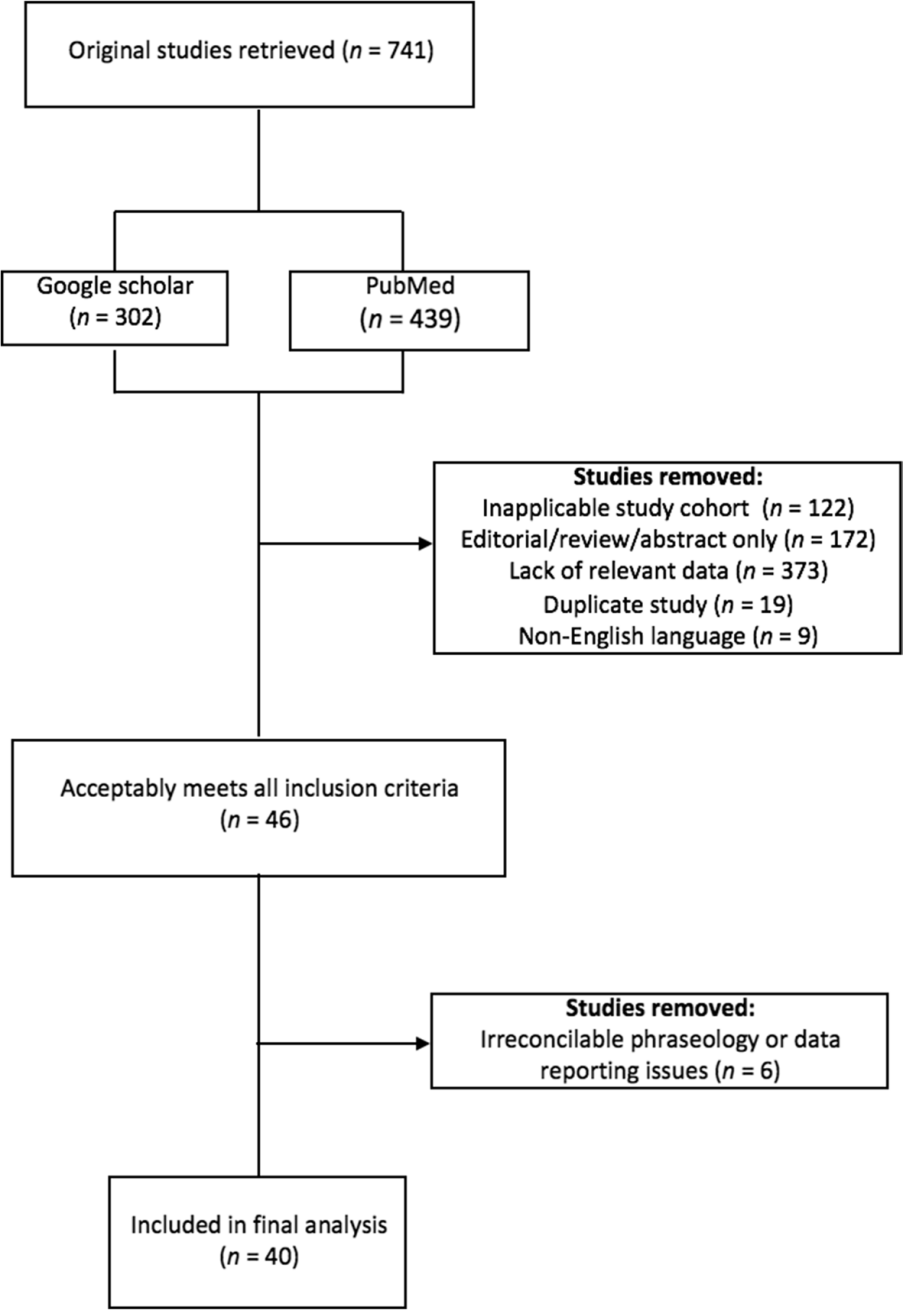
Preferred Reporting Items for Systematic Reviews and Meta-Analyses (PRISMA) flow diagram of included studies

Studies met criteria for inclusion if they satisfied all of the following requirements: (1) they were complete, primary data studies rather than abstracts, meta-analyses, or systematic reviews; (2) they provided relevant data with respect to one or more of the aforementioned guiding research questions; (3) they were published in English; and (4) they contained medical professional or student respondents only; or, if a study included mixed groups with non-medical professionals or students, it segregated and sorted data based on one’s status as a medical professional or non-medical professional. Studies were excluded from further analyses if they failed to meet any one of these four specified requirements (see Fig. 1). There were no fixed search limits regarding year of publication. Overall, out of the 741 studies retrieved in the literature search, 40 studies were identified as meeting all the necessary inclusion criteria (see Table 1). The Cochrane Collaboration Risk of Bias Assessment Tool (version 2) was used to assess the risk of bias for each study (see Table 2) (Higgins et al. 2021). This tool investigates the following primary sources of bias: selection bias, performance bias, detection bias, attrition bias, and reporting bias. The Cochrane Risk of Bias Assessment Tool outlines the following criteria for assessing for risk of bias in studies: sequence generation (selection bias), allocation concealment (selection bias), blinding of participants and personnel (performance bias), blinding of outcome assessment (detection bias), incomplete outcome data (attrition bias), selective outcome reporting (reporting bias) and other potential sources of bias. Each entry was coded as “high risk” and “low risk,” or “unclear” if there was insufficient information to determine potential bias. Several criteria—including allocation concealment, blinding of participants and personnel, and blinding of outcome assessment—unanimously received ratings of “unclear” due to the nature of the survey-based studies under review.

**Table 1:** Tabulated data from each of the studies included in the systematic review

Study	Country (state)	Total # participants	Participant type	Mean age	% male	Q1: Legal MC (% yes)	Q2: Therapeutic Utility (% yes)	Q3: Recreational (%yes)	Q4: Amend Schedule I (% yes)	Q5: Knowledge (% yes)	Q6: Education (% yes)	Q7: Addiction (% yes)
Ablin (2016)	Israel	23	Rheumatologists	45	78	N/A	74	N/A	N/A	26	N/A	N/A
Ananth (2018)	USA	288	Pediatric oncologists	35	15	92	92	N/A	N/A	N/A	N/A	37
Balneaves (2018)	Canada	182	NP’s	N/A	N/A	97	72	N/A	N/A	N/A	76	N/A
Bega (2017)	Multi-national	56	Neurologists	N/A	55	70	N/A	50	52	N/A	93	N/A
Berlekamp (2019)	USA (OH)	319	Pharmacy students	N/A	N/A	N/A	N/A	44	N/A	N/A	N/A	N/A
Braun (2018)	USA	237	Oncologists	N/A	66	N/A	67	N/A	N/A	29	N/A	N/A
Burke (1971)	USA	1586	Med & pharmacy students	N/A	N/A	16	N/A	41	57	66	N/A	N/A
Caligiuri (2018)	USA (IA)	238	Pharmacy students	N/A	29	N/A	N/A	N/A	N/A	16	77	N/A
Carlini (2015)	USA (WA)	494	Mixed professionals	N/A	31	72	74	N/A	72	N/A	96	62
Chan (2017)	USA (CO)	236	Medical students	<30	52	74	86	64	72	N/A	91	88
Charuvastra (2005)	USA	960	Mixed physicians	N/A	N/A	36	N/A	N/A	N/A	N/A	N/A	N/A
Cogswell (2015)	USA (CA)	175	Medical students & social workers	<30	23	N/A	24	N/A	N/A	N/A	N/A	N/A
Crosby (2018)	USA (WA)	120	Healthcare students	<24	11	N/A	N/A	N/A	N/A	63	N/A	N/A
Crowley (2017)	Ireland	565	GP’s	N/A	49	59	69	N/A	N/A	N/A	N/A	N/A
Doblin (1991)	USA	978	Oncologists	N/A	N/A	53	63	N/A	54	70	N/A	N/A
Ebert (2015)	Israel	72	Mixed physicians	51	65	83	79	23	N/A	80	89	N/A
Fitzcharles (2014)	Canada	128	Rheumatologists	45	57	N/A	N/A	N/A	N/A	26	N/A	N/A
Hwang (2016)	USA (MN)	738	Pharmacists	49	N/A	N/A	43	N/A	N/A	10	N/A	49
Jacobs (2019)	Australia	86	Psychiatrists	N/A	57	N/A	86	N/A	N/A	N/A	N/A	58
Karanges (2018)	Australia	640	GP’s	N/A	33	57	44	N/A	N/A	10	N/A	56
Kondrad (2013)	USA (CO)	520	Family physicians	50	56	19	67	30	37	N/A	92	86
Kusturica (2019)	Serbia	316	Medical students	N/A	32	76	82	N/A	N/A	65	N/A	62
Lieff (1973)	USA (MA)	35	Mixed physicians	N/A	N/A	69	N/A	48	N/A	N/A	N/A	N/A
Linn (1989)	USA	303	Mixed physicians	48	87	N/A	N/A	41	N/A	N/A	N/A	N/A
Luba (2018)	USA	426	Mixed medical professionals	49	43	N/A	89	N/A	N/A	N/A	N/A	N/A
Martins (2017)	USA	137	Mixed medical professionals	39	44	N/A	95	N/A	N/A	N/A	N/A	13
Mathern (2015)	Multi-national	776	Mixed physicians	N/A	N/A	83	35	N/A	N/A	N/A	N/A	N/A
Michalec (2015)	USA (DE)	87	Mixed physicians	>50	78	N/A	N/A	N/A	N/A	39	31	29
Mitchell (2016)	Canada	769	Hospital pharmacists	41	41	N/A	55	N/A	N/A	17	N/A	N/A
Moeller (2015)	USA	311	Pharmacy students	N/A	39	59	91	35	59	N/A	90	N/A
Norberg (2012)	Australia	664	GP’s (503) & Nurses (162)	48	21	34	N/A	N/A	N/A	42	97	N/A
Philpot (2019)	USA (MN)	62	Mixed physicians & PA’s	46	57	39	58	N/A	N/A	50	77	N/A
Ricco (2017)	USA (MN)	60	Family Physicians	N/A	N/A	N/A	25	N/A	N/A	5	N/A	58
Schwartz (1997)	USA	1122	Oncologists	47	N/A	28	N/A	15	28	N/A	N/A	N/A
Sideris (2018)	USA (NY)	164	Mixed physicians	N/A	63	71	N/A	N/A	N/A	40	N/A	N/A
Stojanovic (2017)	Serbia	80	Pharmacy students	N/A	N/A	75	91	N/A	N/A	N/A	68	50
Szyliowicz (2019)	USA (CA)	474	Pharmacists	N/A	41	N/A	75	N/A	N/A	18	92	N/A
Uritsky (2011)	USA	194	Hospice medical professionals	47	17	91	86	90	N/A	N/A	N/A	45
Ziemianski (2015)	Canada	426	Mixed physicians	N/A	N/A	85	73	N/A	N/A	N/A	64	N/A
Zylla (2018)	USA (MN)	153	Oncologists	N/A	N/A	N/A	N/A	N/A	N/A	63	85	N/A
TOTAL	N/A	Total: 15,200 Mean: 380 SD: 345 Median: 263 IQR: 419 Range: 1563	N/A	Mean: 43.8^1^ SD: 4.81^2^ Median: 47 IQR: 4 Range: 16	Mean: 41.3^3^ SD: 17.2^4^ Median: 44 IQR: 26 Range: 76	49.9 (25.7)^5^	64.4 (18.7)^5^	36.5 (17.7)^5^	50.5 (15.4)^5^	41.0 (25.3)^5^	86.2 (13.8)^5^	57.8 (18.4)^5^

Note: *GP* general practitioner, *PA* physician’s assistants, *NP’s* nurse practitioners

*Fully-phrased questions: Q1 = Do you believe that physicians deserve the legal right to prescribe cannabis to patients? [i.e. Do you believe that cannabis should be legalized for therapeutic purposes?]; Q2 = Do you believe that cannabis has any therapeutic utility; Q3 = Do you believe that cannabis should be legalized for recreational use?; Q4 = [For US-based papers only] Do you believe that the USA should amend cannabis’ federal status as a schedule 1 controlled substance (the most restrictive classification, asserting that the substance has no accepted medical use)?; Q5 = Do you feel confident in your level of knowledge regarding the health effects of cannabis?; Q6 = Do you desire additional education regarding MMJ and/or do believe that education on (medical) cannabis should be made readily available to medical professionals?; Q7 = Are you concerned about cannabis’ dependence/addiction potential?

^1^Weighted mean for all studies providing an exact integer value for mean age; ^2^ weighted SD

^3^Pooled and weighted percent-male value; ^4^weighted SD in parentheses

^5^Pooled and weighted percent-yes value; weighted SD in parentheses

**Table 2:** Risk of bias assessment for all contributing studies

Study	Random sequence generation (selection bias)	Allocation concealment (selection bias)	Blinding of participants and personnel (performance bias)	Blinding of outcome assessment (detection bias)	Incomplete outcome data (attrition/nonresponse bias)	Selective reporting (reporting bias)
Ablin, et al.	?	?	?	?	?	✓
Ananth, et al.	?	?	?	?	?	✓
Balneaves, et al.	?	?	?	?	?	✓
Bega, et al.	?	?	?	?	?	✓
Berlekamp, et al.	?	?	?	?	?	✓
Braun, et al.	✓	?	?	?	✓	✓
Burke & Marx	?	?	?	?	✓	✓
Caligiuri, et al.	?	?	?	?	?	✓
Carlini, et al.	?	?	?	?	?	✓
Chan, et al.	?	?	?	?	?	✓
Charuvastra, et al.	?	?	?	?	?	✓
Cogswell & Harris	?	?	?	?	✓	✓
Crosby	?	?	?	?	?	✓
Crowley, et al.	?	?	?	?	?	✓
Doblin & Kleiman	?	?	?	?	?	✓
Ebert, et al.	?	?	?	?	?	✓
Fitzcharles, et al.	?	?	?	?	?	✓
Hwang, et al.	?	?	?	?	?	✓
Jacobs, et al.	?	?	?	?	?	✓
Karanges, et al.	?	?	?	?	?	✓
Kondrad & Reid	?	?	?	?	?	✓
Kusturica, et al.	?	?	?	?	✓	✓
Lieff, et al.	✓	?	?	?	?	✓
Linn, et al.	?	?	?	?	?	✓
Luba, et al.	?	?	?	?	?	✓
Martins , et al.	?	?	?	?	?	?
Mathern, et al.	?	?	?	?	?	✓
Mitchell, et al.	?	?	?	?	?	✓
Moeller & Woods	?	?	?	?	✓	✓
Norberg, et al.	?	?	?	?	?	✓
Philpot, et al.	?	?	?	?	?	✓
Rapp, et al.	?	?	?	?	?	✓
Ricco, et al.	?	?	?	?	?	?
Schwartz, et al.	?	?	?	?	?	✓
Sideris, et al.	?	?	?	?	?	✓
Stojanovic, et al.	?	?	?	?	✓	✓
Szyliowicz, et al.	?	?	?	?	?	?
Uritsky, et al.	?	?	?	?	?	✓
Ziemianski, et al.	?	?	?	?	?	✓
Zylla, et al.	?	?	?	?	?	✓

**Key:**

? = unclear risk

X = high risk

✓ = low risk

Data from studies which met all the inclusion criteria were subsequently logged and organized on a master spreadsheet. While sorting through papers to extract data pertaining to the seven guiding research questions, it became apparent that while most papers addressed similar topics, they often phrased their questions in slightly different ways. For example, Chan et al. (2017) asked respondents to either agree or disagree with the following statement: “physicians should recommend marijuana as medical therapy,” whereas other researchers, such as Ananth et al. (2018), asked respondents to state whether or not they—as physicians—would be willing to prescribe marijuana to a patient (Ananth et al. 2018; Chan et al. 2017). Although these survey questions may not be exactly analogous, they both address the question of whether or not physicians should be allowed to authorize MC. Therefore, it was determined that both questions could be coded under the same category—in this instance, both were included in analyses related to research question 1: “Do healthcare professionals believe that cannabis should be legalized for medicinal purposes?” Similar judgment calls were made in numerous other instances when the phraseology of certain studies did not directly align with the phraseology used in the spreadsheet. A full layout of the phraseological sorting process—in accordance with the seven guiding research questions—is provided in Appendix B.

### Statistical analyses

Chi-square tests were used to assess the presence or absence of statistically significant differences between the two main survey cohorts: medical students and medical professionals. For these analyses, the total number of individual respondents from all the relevant studies who reported either “yes” or “no” to each research question were pooled into groups; then, a chi-square analysis determined if there were significant differences in the relative frequencies between each group. Additionally, Pearson’s *r* bivariate correlations were used to assess any relevant differences in entire studies’ responses to the research questions over time (i.e. by the year of the study’s publication). Furthermore, some temporal analyses (all using Pearson’s *r* bivariate correlations) assessed respondents’ opinions regarding MC with respect to the number of years preceding or following MC legalization in the state or country of the study’s publication. For these analyses, the year of MC legalization was identified for each state or country and labeled as “year 0”; then, the year of MC legalization was subtracted from the year of the study’s publication to yield the number of years distancing the study from the year of MC legalization. Finally, some temporal analyses featured a preponderance of studies conducted in a truncated time period—with only a few outlying studies published many years apart from the central cohort; in these instances, the outliers were excluded from analysis.

## Results

### Descriptive statistics

In total, 40 studies provided data which were included in the final analyses; 26 studies were conducted exclusively in the USA, and the other 14 were conducted either multi-nationally or in countries other than the USA. The 40 studies produced a pool of exactly 15,200 respondents, yielding a mean of 380 respondents per study (SD = 345). For the studies which reported such statistics, there was an overall mean age of 43.8 years (SD = 4.81), with 41.3% of respondents being male (SD = 17.2). Overall, 8 studies surveyed students only (20%), 31 surveyed medical professionals only (78%), and 1 surveyed both students and professionals (3%). Within the group of studies that only surveyed medical professional, 5 surveyed pediatric or adult oncologists (16%), 4 surveyed family physicians or general practitioners (13%), 3 surveyed pharmacists (10%), 2 surveyed rheumatologists (6%), 1 surveyed psychiatrists (3%), 1 surveyed exclusively nurse practitioners (3%), 1 surveyed hospice professionals (3%), 1 surveyed neurologists (3%), and 13 surveyed a mixed cohort of various medical professionals (42%). All studies included in the final analyses were evaluated for risk of bias according to the Cochrane Collaboration. While a preponderance of studies had an unclear risk of bias, none clearly expressed a high risk of bias which could threaten the review’s overall findings or conclusions (see Table 2, above).

### Question 1: Do you believe that cannabis should be legalized for therapeutic purposes?

**Fig. 2:**
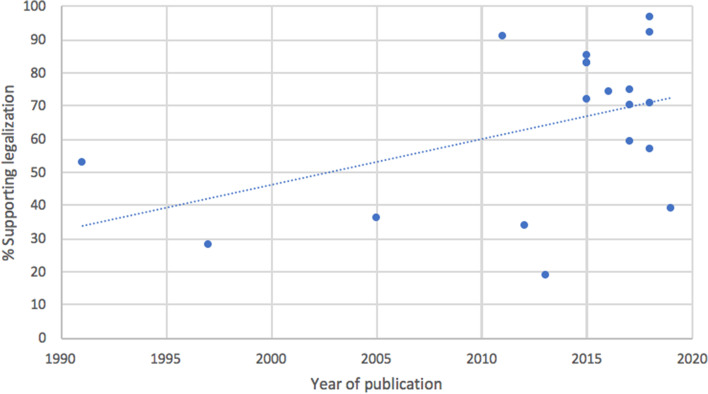
Medical students’ and professionals’ attitudes towards MC legalization, by year of study publication. Explanatory legend: Study question 1; 2 pre-1990 outliers removed; 21 studies total; *r*(19) = .44, *p* = .045

An analysis of question 1 drew data from 21 studies (8016 respondents) published between 1991 and 2019. A Pearson’s bivariate correlation between a study’s year of publication and the percentage of respondents supporting MC legalization suggests that both medical students’ and professionals’ support for the legalization of MC increased over time (*r*(19) = .44, *p* = .045; Fig. 2). Out of the entire sample, 49.9% of all respondents favored legalization (SD = 25.7, range: 16–97%). The same correlational analysis amongst only medical professionals (following the removal of the 4 student-only studies) from studies published between 1991 and 2019 did not reach statistical significance (*r*(15) = .42, *p* = .093). Additionally, a correlational analysis between the number of years following or preceding MC legalization in the state or country of a study’s publication (within ±20 years) and the percentage of respondents supporting MC legalization did not meet statistical significance (*r*(7) = .53, *p* = .143).

A chi-square test comparing students’ (*N* = 1911, 5 studies) attitudes towards the legalization of MC against those of medical professionals (*N* = 7108, 18 studies) revealed a significant difference between the two cohorts, with medical professionals favoring legalization at a significantly higher rate than students (52% vs. 42%, respectively; *χ*^2^ (1, *N* = 9019) = 50.72, *p* < .001). Finally, a cross-national comparison of respondents’ attitudes regarding the legalization of MC reveals that levels of support markedly vary between countries; Canada demonstrated the greatest support for the legalization of MC (89%, *N* = 608, 2 studies), followed by Israel (83%, *N* = 71, 1 study), Serbia (76%, *N* = 396, 2 studies), Ireland (59%, *N* = 565, 1 study), and Australia (45%, *N* = 1304, 2 studies), while the USA demonstrated the least support for the legalization of MC (42%, *N* = 5853, 13 studies).

### Question 2: Do you believe that cannabis has any therapeutic utility?

An analysis of research question 2 drew data from 26 studies (9,265 total respondents) and assessed respondents’ belief in cannabis’ medical utility. Out of the entire sample, 64.4% of all respondents espoused belief in cannabis’ therapeutic utility (SD = 18.7). A chi-square test comparing medical students’ (*N* = 1118, 5 studies) versus medical professionals’ (*N* = 7589, 21 studies) belief in cannabis’ medical utility yielded a significant difference, with students reporting greater confidence in cannabis’ medical utility than medical professionals (77% vs 65%, respectively; *χ*^2^ (1, *N* = 8707) = 62.72, *p* < .001). Additionally, a cross-national comparison of respondents’ belief in cannabis’ therapeutic utility revealed that levels of belief markedly vary between countries; Serbian respondents reported the highest rates of belief in cannabis’ medical utility (84%, *N* = 396, 2 studies), followed by Israel (82%, *N* = 95, 2 studies), the USA (70%, *N* = 5320, 16 studies), Ireland (68%, *N* = 565, 1 study), and Canada (63%, *N* = 1353, 3 studies), while Australian respondents reported the lowest rates of belief in cannabis’ medical utility (49%, *N* = 726; 2 studies).

### Question 3: Should cannabis be legalized for recreational use?

An analysis of research question 3 drew data from 11 studies (4754 total respondents) published between 1971 and 2019 and assessed whether medical students’ and professionals’ attitudes towards the legalization of recreational cannabis have changed over time. A Pearson’s correlation between year of publication and the proportion of respondents who support recreational legalization revealed no statistically-significant relationship (*r*(9) = .11, *p* = .746). Out of the entire sample, 36.5% of all respondents believed cannabis should be recreationally legalized (SD = 17.7). A chi-square test of medical students’ (*N* = 1834, 4 studies) versus medical professionals’ (*N* = 2302, 7 studies) support for recreational legalization yielded a statistically-significant difference, with students demonstrating greater support for recreational legalization than medical professionals (43% vs. 30%, respectively; *χ*^2^ (1, *N* = 4136) = 78.88, *p* < .001).

### Question 4: Should the US federal government amend cannabis’ Schedule I status?

An analysis of research question 4 drew data from 8 studies (5303 total respondents) and assessed US-based respondents’ opinions regarding the federal rescheduling of cannabis. Out of the entire sample, 50.5% of all respondents believed that the US federal government should amend cannabis’ Schedule I status (SD = 15.4). A chi-square test between medical students (*N* = 1204, 2 studies) and professionals (*N* = 3045, 5 studies) yielded a significant difference between each group’s level of support for the federal rescheduling of cannabis, with students supporting more lenient federal regulations at a higher rate than professionals (60% vs. 46%, respectively; *χ*^2^ (1, *N* = 4249) = 70.76, *p* < .001).

### Question 5: Are you confident in your level of knowledge regarding the health effects of cannabis?

**Fig. 3:**
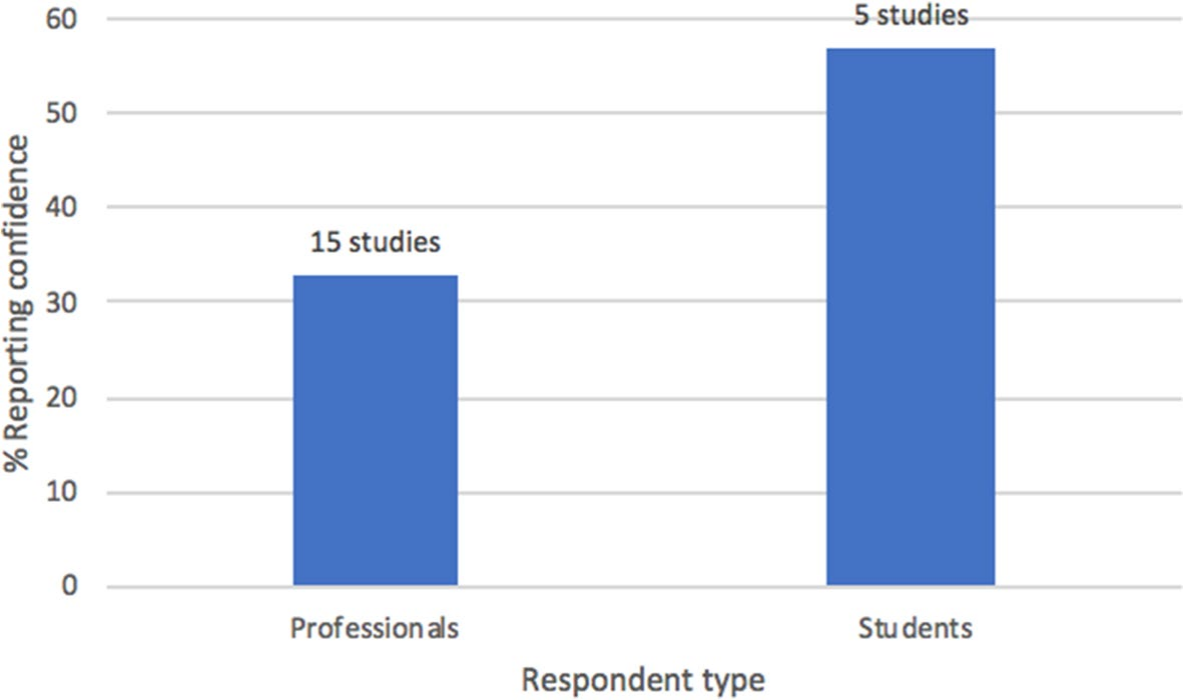
Respondents’ self-reported confidence in their knowledge of MC, by respondent type. Explanatory legend: Medical professionals (*N* = 5068) vs. students (*N* = 1642) (*χ*^2^ (1, *N* = 6710) = 325.19, *p* < .001)

An analysis of research question 5 drew data from 19 studies (7,509 respondents) and evaluated respondents’ self-reported level of confidence regarding their knowledge of cannabis and its health effects. A Pearson’s correlation between the amount of years following or preceding MC legalization in the state or country of a study’s publication (limit: ±20 years) and respondents’ self-reported level of confidence revealed no statistically significant relationship (12 studies, *r*(10) = .22, *p* = .485). Out of the entire sample, 41.0% of all respondents espoused confidence in their knowledge of cannabis’ health effects (SD = 25.3, range: 5–80%). A chi-square analysis of respondents’ self-reported knowledge by respondent type (medical professionals [*N* = 5068, 15 studies] vs. students [*N* = 1642, 5 studies]) revealed significant differences between the two cohorts (*χ*^2^ (1, *N* = 6710) = 325.19, *p* < .001; see Fig. 3). Overall, students reported the greatest confidence in their self-reported knowledge of MC, with medical professionals (on average) reporting significantly lower rates of confidence regarding their knowledge of MC (58% vs. 33%, respectively). Finally, a cross-national comparison of respondents’ self-reported confidence in their knowledge of MC revealed that levels of knowledge markedly vary between countries; Israeli respondents reported the highest rates of self-reported confidence in their knowledge of MC (67%, *N* = 94, 2 studies), followed by Serbia (65%, *N* = 316, 1 study), the USA (45%, *N* = 4125, 12 studies), and Australia (26%, *N* = 1300, 2 studies), while Canadian respondents reported the lowest rates of self-reported confidence in their knowledge of MC (18%, *N* = 876, 2 studies).

### Question 6: Should there be more educational material available regarding MC?

An analysis of research question 6 drew data from 15 studies (4055 total respondents) and assessed whether respondents desired more education about MC, and if they believed that information about MC should be incorporated into medical school curricula. Out of the entire sample, 86.2% of respondents believed that there should be more educational material available on MC (SD = 13.8)—with 12 out of the 15 studies reporting 75% or more of respondents desiring further education. A Pearson’s correlation between the year of a study’s publication (range: 2012–2019) and the percentage of respondents’ espousing a personal or general desire for more knowledge regarding MC did not meet statistical significance, implying no significant differences over time (*r*(13) = − .10, *p* = .713).

### Question 7: Are you concerned about cannabis’ dependence/addiction potential?

Lastly, an analysis of research question 7 drew data from 13 studies published between 2011 and 2019 (3876 total respondents) and asked respondents if they were concerned about MC’s potential to cause addiction or dependence in patients. Out of the entire sample, 57.8% of all respondents were concerned with cannabis’ potential to cause addiction or dependence (SD = 18.4). A Pearson’s bivariate correlation between the year of a study’s publication (range: 2011–2019) and the percentage of respondents espousing concern regarding MC’s potential to cause addiction or dependence and did not meet statistical significance, suggesting no change over time (*r*(12) = − .12, *p* = .678).

## Discussion

This systematic review provided a multi-variate analysis of the existing literature on medical professionals’ and students’ attitudes and knowledge regarding medicinal cannabis (MC). Most notably, it was found that both medical students’ and professionals’ support for the legalization of MC has significantly increased throughout the last three decades, in addition to medical professionals being more likely than students to endorse MC legalization (52% vs. 42%, respectively). Furthermore, respondents consistently reported a strong desire for more education about MC, and a substantial concern regarding MC’s potential to cause dependence and addiction. Pearson’s correlations between year of publication and survey responses for both of these queried variables suggest minimal changes over time (2011–2019 for addiction and dependence, 2012–2019 for additional education). Lastly, support for the legalization of MC, respondents’ self-reported confidence regarding their knowledge of MC, and respondents’ belief in cannabis’ medical utility all showed considerable differences between countries. More broadly, the analyses conducted in this review sought to address each of the following guiding research questions.

### Question 1: Should cannabis be legalized for therapeutic purposes?

Question 1 assessed respondents’ support for the legalization of MC. It was expected that support for the legalization of MC would increase over time due to ongoing sociocultural and legislative trends favoring legalization—which may serve to reduce stigma and increase the normalization of cannabis within the medical community. Moreover, results from Gardiner et al.’s (2019) systematic review indicate that newer studies tend to yield more accepting attitudes towards MC. Results from this systematic review supported the hypothesis, as respondents’ level of support for the legalization of MC was shown to significantly increase from 1991 to 2019 (see Fig. 2). Also, it was expected that students would demonstrate greater support for MC legalization compared to medical professionals, given the premise that many professionals might have been educated during an era in which cannabis was largely demonized in society and the medical community, and also given the established research finding that (at least within the USA) younger individuals are adopting more permissive views towards cannabis (Schmidt et al. 2016). However, results from this systematic review actually indicated the reverse, with medical professionals demonstrating greater support for MC legalization than students. This finding could be explained by entertaining the notion that students may want to espouse more orthodox viewpoints during their educational years, so as not to appear overly progressive and radical, which could possibly jeopardize their clinical accreditation. Also, a majority of existing educational programs underrepresent the medical value of cannabis and cannabinoids and instead emphasize the risks and side effects, such as addiction and dependence. This extant curricular bias could help explain students’ concerns and lack of support for MC.

Furthermore, it was hypothesized that respondents’ support for the legalization of MC would be highest in countries that took early legislative steps to legalize MC, due to the established research finding that the passage of MC laws tends to correlate with more lenient views towards cannabis—especially amongst younger people (Schmidt et al. 2016). This expectation was largely confirmed, as Canadian respondents demonstrated the greatest support for the legalization of MC (89%), while US respondents demonstrated the least support for the legalization of MC (42%); Canada legalized MC nationwide in 2001, while several states within the USA still fully prohibit the medical prescription of cannabis. However, this presumption has been challenged by Gritsenko et al.’s 2020 paper investigating the effect of religion on Russian medical students’ attitudes towards the legalization of MC (Gritsenko et al. 2020). They found that 80% of non-religious students supported legalization, compared to 60% of religious students. Despite the limited sample size of 828 students, these numbers indicate greater support for MC legalization in Russia—where all forms of cannabis are criminalized—compared to the USA. Notwithstanding, the results from Gritsenko et al. (2020) support the hypothesis that medical students, and younger people in general, would express more lenient views towards MC legalization.

### Question 2: Do you believe that cannabis has any therapeutic utility?

Question 2 investigated respondents’ belief in cannabis’ medical utility. It was hypothesized that students would express greater faith in cannabis’ medical utility under the premise that students (being younger, on average, than professionals) would be more likely to have been raised in a sociopolitical climate more accepting of cannabis’ medical applications. This hypothesis was supported by the data, with 77% of students expressing belief in cannabis’ medical utility as opposed to only 65% of medical professionals. Similarly, it was anticipated that respondents’ from countries with a longstanding legal acceptance of MC would espouse greater confidence in cannabis’ medical utility; however, the data did not support this hypothesis, as Serbian respondents reported the greatest belief in cannabis’ medical utility (84%) despite the fact that MC remains illegal in Serbia, while Australian respondents reported the lowest levels of belief in cannabis’ medical utility (49%), despite the fact that Australia federally legalized MC in 2016.

### Question 3: Should cannabis be legalized for recreational use?

Research question 3 assessed respondents’ support for the recreational legalization of cannabis. It was expected that support for recreational legalization would increase over time due to ongoing sociopolitical trends favoring the decriminalization and legalization of recreational cannabis. However, no significant correlational trends were observed over time. The data indicates that approximately one in two respondents (i.e., 50%) favor the legalization of recreational cannabis—regardless of the year of the study’s publication. One possible explanation is that recreational legalization has blossomed in recent years without a commensurate development in cannabis-related medical education. Moreover, several researchers have reported spikes in cannabis-related hospitalizations following recreational legalization (Auger et al. 2020; Zvonarev et al. 2019). Therefore, despite the general public’s more lenient views towards recreational legalization in recent years, physicians may continue to harbor reservations—especially as cannabis-related hospitalizations climb in the absence of proportionate developments in cannabis-related medical research. Likewise, it was expected that students would express greater support for recreational cannabis, under the premise that younger respondents tend to hold more permissive views toward cannabis regulation (Schmidt et al. 2016). The results supported this hypothesis, as 43% of students reported support for recreational legalization, as opposed to only 30% of medical professionals.

### Question 4: Should the US federal government amend cannabis’ Schedule I status?

Research question 4 assessed US-based respondents’ opinions regarding the federal rescheduling of cannabis. Once again, it was expected that students would express greater support for the federal rescheduling of cannabis, under the premise that younger respondents tend to espouse more permissive views towards cannabis regulation (Schmidt et al. 2016). The data supported this hypothesis, with 60% of students indicating support for more lenient federal restrictions of cannabis use, as opposed to only 46% of medical professionals.

### Question 5: Are you confident in your level of knowledge regarding the health effects of cannabis?

Question 5 assessed respondents’ self-reported confidence regarding their knowledge of MC. Results from Gardiner et al.’s (2019) review indicate that healthcare professionals consistently report low levels of self-perceived knowledge regarding MC; notwithstanding, it was hypothesized that confidence levels would rise as the number of years following MC legalization in the country of a study’s publication increased, due to respondents’ from those countries having an increased likelihood of being exposed to cannabis in clinical settings. However, no statistically significant correlational relationship was observed. Moreover, it was expected that professionals (as opposed to students) would express greater confidence in their knowledge of MC, given their more extensive medical training and clinical experience; however, the opposite result was observed, with 58% of students reporting an adequate (or better) knowledge of MC and just 33% of medical professionals reporting an adequate (or better) knowledge of MC (see Fig. 3). This finding could be the result of students—and younger respondents in general—having more lenient attitudes towards cannabis, resulting in a greater perceived sense of knowledge about MC; or, it could be a manifestation of the Dunning-Kruger effect, a cognitive bias in which individuals with an inferior understanding of a concept tend to overestimate their own perceived level of knowledge (Kruger and Dunning 1999). Notably, results from this study do not directly align with results from other reviews on this topic. Gardiner et al. (2019) found self-reported knowledge to be low amongst all types of health professionals, while Zolotov et al. (2021) found that health students overwhelmingly lacked knowledge and confidence in counseling patients on MC (Gardiner et al. 2019; Zolotov et al. 2021). Therefore, this study is the first to report significant differences in self-reported confidence levels between healthcare students and professionals. More in-depth studies are needed to survey levels of self-reported confidence in MC knowledge, particularly amongst healthcare students, to help elucidate the discrepancies between this study and the results of Zolotov et al. (2021).

Furthermore, it was hypothesized that respondents from countries with a longstanding legal acceptance of MC would demonstrate greater levels of confidence regarding their knowledge of MC, under the premise that respondents’ from such countries would have an increased likelihood of being exposed to cannabis in clinical settings. The data largely supported this hypothesis, with Israeli respondents (where MC has been legal since 1973) reporting the greatest levels of confidence (67%) and Canadian respondents (where MC has only been legal since 2001) reported the lowest levels of confidence (18%) (Wilkinson and Tarnopolsky 2019).

### Question 6: Should there be more educational material available regarding MC?

Question 6 assessed respondents’ desire for more educational material regarding MC—including supplemental educational programs for professionals and the incorporation of cannabis-related material into the existing medical school curriculum. Results from Gardiner et al.’s (2019) review found that many health professionals desired more education regarding MC, and it was expected that respondents from more recent studies would express an increased desire for further education, given the heightened acceptance of cannabis as a legitimate medical therapy in recent years. However, a Pearson’s bivariate correlation revealed no significant change in respondents’ desire for more education between 2012 and 2019, and the data actually indicated an apparent ceiling effect—with around 80% of respondents desiring more educational material regardless of the year of the study’s publication.

### Question 7: Are you concerned about cannabis’ dependence/addiction potential?

Lastly, research question 7 asked respondents if they were concerned about MC’s potential to cause addiction or dependence in patients. While Gardiner et al.’s (2019) review did not directly address issues of addiction and dependence, they found that many health professionals raised concerns regarding adverse psychiatric effects. For this review, it was expected that older studies would reflect greater levels of concern, given the established research finding that the perceived harmfulness of cannabis has decreased significantly since 1991 (Keyes et al. 2016). However, a Pearson’s bivariate correlation revealed no significant change between 2011 and 2019, with approximately one in two respondents (i.e., 50%) expressing concern for MC’s addiction and dependence potential regardless of the year of the study’s publication.

## Limitations

It is important to note that this systematic review was affected by several identifiable limitations. Firstly, there was significant variability between the individual studies, including: incongruency in the survey methods and individual phraseologies used in data collection; differences in cannabis regulatory policy in the states and countries in which the surveys were conducted; and differences in the proportions of the types of respondents who answered the surveys (i.e., physicians, pharmacists, and nurses). For instance, many studies included cohorts of medical professionals who specialized in a variety of subfields (e.g., neurology, pharmacy, oncology, and rheumatology); therefore, the analyses presented in this systematic review are generalized findings that combine the responses of all medical professional subtypes. This necessary methodological procedure led to the overall generalization of the medical professional cohort, consequently nullifying any potential differences or distinctions within the overarching “medical professional” group. In addition, far more studies assessed the opinions of medical professionals (31 studies) as opposed to those of medical students (9 studies), which limits the strength of the comparisons made between the two cohorts. Relatedly, not enough studies analyzed in this review published data on relevant covariates (e.g., gender, religiosity, political affiliations, etc.) to analyze data along these variables. Another very important consideration is whether respondents’ personal use of either medicinal or recreational cannabis biased or shaped their opinions. As more studies emerge, research should strive to better understand how these numerous covariates influence respondents’ opinions toward MC.

Given the rapidly increasing interest in the field of MC, it is also crucial to note that the literature search was performed roughly halfway through 2019, resulting in the exclusion of numerous, relevant studies which were published afterwards. Such studies include: Gritsenko et al. (2020), Benavides et al. (2020), and Arnfinsen and Kisa (2021), among others (Arnfinsen and Kisa 2021; Benavides et al. 2020; Gritsenko et al. 2020). The reason for the 2019 search cutoff is due to the fact that this study was conducted as part of an undergraduate thesis completed in December 2019. Therefore, the authors of this review recommend that follow-up studies be performed in the coming years to draw temporal comparisons to the results of the present study. As research within the field continues to greatly proliferate, these follow-ups will help reveal distinct trends and key ways in which attitudes and knowledge are shifting so that medical professionals, educators, and policymakers can stay up-to-date with respect to rapidly changing developments within the field.

Also, while the 40 studies provided enough data to conduct a meaningful systematic review, most did not provide the necessary metrics (e.g., pre/post comparisons and between-groups comparisons) required to perform an even more comprehensive meta-analysis. Going forward, more studies should begin to yield the requisite effect sizes required to perform meta-analyses as the surveys used in these studies begin to include more data pertaining to mediation analyses and pre/post comparisons. Lastly, a major preponderance of studies collected for this systematic review were published after 2010 (34 out of 40), which limits the statistical power of long-term temporal analyses—resulting in a reduced range of years in which comparisons can be made to assess changes in knowledge and attitudes over time.

## Implications

Crucially, results from this systematic review have important implications for the continued adoption of MC within the global medical community. Notably, this review found that medical students are significantly more likely to report high levels of confidence regarding their knowledge of MC as compared to medical professionals. Consequently, establishing an objective set of scientifically sound research and educational protocols regarding the management of MC will be imperative in mitigating potential barriers which might arise between more orthodox, senior clinicians and younger, more progressive clinicians as MC and other alternative therapies increasingly augment the conventional medical canon. In addition, respondents’ consistently expressed concerns regarding MC’s potential to induce addiction and dependence—with about one-half of all respondents espousing concern regardless of the year in which the study was conducted. Accordingly, future research and educational programs should specifically address the risks of addiction and dependence to better inform the medical community on the potential risks of MC prescription. Taken together, these data will help to inform future clinical investigations and further scholarship into MC-related topics, particularly in regions where MC is just beginning to come into clinical usage. Thankfully—through the analysis, elucidation, and dissemination of past and ongoing trends pertaining to the progression of MC acceptance in clinical communities—it is now possible for future practice and policy to become more streamlined, safe, and effective.

## Conclusion

This systematic review assessed contemporary and relevant trends pertaining to medical professionals’ and students’ opinions and knowledge regarding medicinal cannabis (MC). Moreover, this review expanded upon Gardiner et al. (2019) and Zolotov et al.’s (2021) similar reviews by (a) examining more studies from a broader array of countries, (b) investigating highly specific research questions, (c) exploring temporal trends in the data, (d) comparing student and professional cohorts, and (e) performing statistical analyses which yielded significant trends pertaining to medical students’ and professionals’ knowledge and attitudes regarding MC. Most importantly, the finding that both medical students’ and professionals’ acceptance of MC has significantly increased in recent decades—in conjunction with their consistent, strong desire for more educational material—suggests that the medical community should prioritize the development of MC educational programs. MC is far more likely to succeed as a safe and viable therapy if the medical professionals who administer it are well-trained and confident regarding its clinical effects. Notwithstanding, the preponderance of highly restrictive legislative policies limiting cannabis’ status as a subject of scientific inquiry has led to a dearth of educational material on MC. Therefore, results from this systematic review should encourage the medical community to more seriously consider relevant policy work along with honest, comprehensive investigations into MC to assuage the ongoing stigma and misinformation currently surrounding it—which will help facilitate its safe and effective integration into commonly-accepted medical practice.

## Data Availability

Full methodology is available at the PROSPERO database (https://www.crd.york.ac.uk/prospero/) under the registration number CRD42020204382. Additionally, appendices A and B provide extensive search and coding methodology, and the data pulled from each selected article is available in Table 1.

## Appendix A

### Search strategy for PubMed

1. **Respondent type**: (“physician” OR “health professional” OR “pharmacist” OR “pharmacy” OR “providers” OR “students” or “doctors” OR “oncologist” OR “rheumatologist” OR “clinicians” OR “nurses”).
  AND
2. **Solicitation type**: (“attitudes” OR “opinion” OR “survey” OR “perspectives” OR “knowledge” OR “beliefs”).
  AND
3. **Substance type:** (“medical cannabis” OR “cannabis” OR “marijuana” OR “medical marijuana” OR “cannabinoids” OR “psychoactive”)

Search strategy for Google Scholar

1. **Respondent type**: (“physician” OR “physicians” OR “medical professional” OR “pharmacist” OR “pharmacy” OR “providers” OR “healthcare” OR “students” or “doctors” OR “oncologist”).
  AND
2. **Solicitation type**: (“attitudes” OR “opinion” OR “survey” OR “perspectives” OR “knowledge” OR “perceptions” OR “support”).
  AND
3. **Substance type:** (“medical cannabis” OR “drug” OR “marijuana” OR “medical marijuana” OR “cannabinoid” OR “psychoactive”)

## Appendix B

Analysis and sorting by research question phraseology:

- Research question 1: Do you believe that physicians deserve the legal right to prescribe cannabis to patients? (i.e. Do you believe that cannabis should be legalized for therapeutic purposes?)

Qualified survey questions included the following phrases:

- “Doctors should be able to legally prescribe marijuana as medical therapy” (Charuvastra et al. 2005; Philpot et al. 2019).
- “Doctors should recommend medical marijuana (MMJ; as medical therapy)?” (Chan et al. 2017; Kondrad and Reid 2013).
- “Marijuana should be made available by prescription”) (Doblin and Kleiman 1991; Jacobs et al. 2018; Schwartz et al. 1997; Uritsky et al. 2011).
- “Cannabis should be legalized/available for medicinal purposes” (Bega et al. 2016; Crowley et al. 2017; Mathern et al. 2014; Norberg et al. 2012; Sideris et al. 2018).
- “Clinicians should be able to authorize MC without fear of legal action” (Carlini et al. 2016).
- “MMJ should be legalized in all states” (Moeller and Woods 2015).
- “Specialist physicians should have authority to prescribe CTP” (Balneaves et al. 2018; Ziemianski et al. 2015).
- “The use of CTP should be legalized/approved in Serbia” (Kusturica et al. 2019; Stojanovic et al. 2017).
- “MD’s should play a role in MMJ authorization” (Ebert et al. 2015).
- “MJ should be legalized provided it is under medical supervision” (Burke and Marx 1971).
- “There should be some form of legalized marijuana use" (Lieff et al. 1973).
- “Are you willing to help patients access MMJ?” (Ananth et al. 2018).

- Research question 2: Do you believe that cannabis has any therapeutic utility?

Qualified survey questions included the following phrases:

- “If marijuana were legally available, I would recommend the use of marijuana to a patient” (filed under the survey subscale “belief that marijuana has medical benefits”) (Chan et al. 2017).
- I am concerned that there is limited evidence demonstrating cannabis’ medical efficacy (Those who reported “low concern” were interpreted as espousing belief in MC’s utility) (Ricco et al. 2017).
- “Marijuana helps patients who suffer from chronic, debilitating medical conditions” (Carlini et al. 2016; Ebert et al. 2015; Kondrad and Reid 2013).
- “Do you believe that MMJ can help prevent nausea and vomiting (in patients receiving chemotherapy or radiation)?” (Braun et al. 2018; Doblin and Kleiman 1991; Luba et al. 2018).
- “Do you approve of using MMJ to help manage patients’ symptoms?” (Ananth et al. 2018).
- “Do you think MMJ has medical benefits/efficacy?” (Mitchell et al. 2016; Szyliowicz and Hilsenrath 2019; Uritsky et al. 2011).
- “Do you believe legalization [of cannabis] would be medically efficacious?” (Cogswell and Harris 2015).
- “Do you believe that MC is a legitimate medical therapy?” (Philpot et al. 2019).
- “Do you recognize MMJ as an oncological therapy?” (Moeller and Woods 2015).
- “Do you have a patient who you agree would benefit from medical cannabis?” (Karanges et al. 2018).
- “Are you certain about MMJ’s therapeutic value?” (Auger et al. 2020; Ziemianski et al. 2015).
- “Cannabis has a role in palliative care” (Crowley et al. 2017).
- “Assess your concern regarding the limited evidence of therapeutic benefits from MMJ” (1-7 Likert scale [1 = least concern, 7 = most concern]; responses of 1-3 were approved and consolidated for analyses reporting “confidence in cannabis’ medical efficacy”) (Hwang et al. 2016).
- “Do you believe [medical] marijuana/CBD has efficacy in treating (childhood) epilepsy?” (Ablin et al. 2016; Hwang et al. 2016; Mathern et al. 2014).
- “Do you believe that marijuana has an acceptable role in medicine?” (Martins-Welch et al. 2017).
- “I am familiar with the possible therapeutic effects of cannabis” (Kusturica et al. 2019).
- “Do you agree that cannabis and its derivatives could potentially have therapeutic effects?” (Stojanovic et al. 2017).

- Research question 3: Do you believe that marijuana should be legalized for recreational use?

Qualified survey questions included the following phrases:

- “(Do you believe that) marijuana should be legalized for recreational use?” (Berlekamp et al. 2019; Chan et al. 2017; Kondrad and Reid 2013; Moeller and Woods 2015; Schwartz et al. 1997).
- “What legal action should be taken for the possession of marijuana: 1) No legal action; 2) Citation with a fixed fine; 3) Misdemeanor; 4) Felony? (Linn et al. 1989).
- “Marijuana should be regulated in the same way as alcohol” (Lieff et al. 1973).
- “Should cannabis be made recreational?” (Bega et al. 2016).
- “Are you in favor of legalizing cannabis for non-medical purposes?” (Ebert et al. 2015).
- “Free access should be granted for the use of marijuana” (Burke and Marx 1971).
- “All marijuana should be legalized” (Uritsky et al. 2011).

- Research question 4: [For US-based papers only] Do you believe that the USA should amend cannabis’ federal status as a Schedule 1 controlled substance (the most restrictive classification, asserting that the substance has no accepted medical use)?

Qualified survey questions included the following phrases:

- “Do you favor the Drug Enforcement Agency (DEA) reclassifying marijuana so that it is no longer a Schedule 1 drug?” (Bega et al. 2016; Chan et al. 2017; Kondrad and Reid 2013).
- “Do you support the rescheduling of marijuana to permit its use in medicine?” (Doblin and Kleiman 1991; Schwartz et al. 1997).
- “Cannabis should be rescheduled so that it is no longer a Schedule 1 drug with no medical benefits” (Carlini et al. 2016).
- “Do you favor change in (federal) marijuana control?” (Burke and Marx 1971).

- Research question 5: Do you feel confident in your level of knowledge regarding the health effects of cannabis?

Qualified survey questions included the following phrases:

- “Do you feel confident in your ability to prescribe marijuana, or would you require more knowledge before prescribing?” (Doblin and Kleiman 1991).
- “Self-reported knowledge of cannabis” (reported on a 1-5 Likert scale; responses of 3, 4, or 5 [acceptable, strong, and very strong knowledge, respectively] were approved and consolidated for analyses reporting “confidence in knowledge of medicinal cannabis”) (Norberg et al. 2012).
- “Self-reported competency regarding knowledge of MMJ efficacy” (1-5 Likert scale; responses of 4 or 5 [strong and very strong knowledge, respectively] were approved and consolidated for analyses reporting “confidence in knowledge of medicinal cannabis”) (Ricco et al. 2017).
- “How much knowledge do you have about medical marijuana?” (6 categories: very little knowledge, some knowledge, moderate knowledge, substantial knowledge, high level of knowledge, and professional level of knowledge; “substantial knowledge,” “high level of knowledge,” and “professional level of knowledge” were approved and consolidated for analyses reporting “confidence in knowledge of medicinal cannabis”) (Szyliowicz and Hilsenrath 2019).
- “Do you feel adequately prepared to answer patients’ questions about MMJ?” (Philpot et al. 2019)
- “Do you consider yourself well-informed about the endocannabinoid system?” (Sideris et al. 2018).
- “Do you consider yourself knowledgeable about MMJ therapy?” (Mitchell et al. 2016; Rapp et al. 2015).
- “Confidence in discussing risks and benefits of medical cannabis” (4 categories: very confident; somewhat confident; somewhat not confident, not at all confident; “very confident” and “somewhat confident” responses were approved and consolidated for analyses reporting “confidence in knowledge of medicinal cannabis”) (Zylla et al. 2018).
- “I have good knowledge around the (side) effects of medicinal cannabis” (Karanges et al. 2018; Kusturica et al. 2019).
- “I know how to talk to providers about the risks and benefits of MMJ use” (“confident” and “somewhat confident” responses were approved and consolidated for analyses reporting “confidence in knowledge of medicinal cannabis”) (Caligiuri et al. 2018).
- “Do you feel sufficiently knowledgeable to make recommendations regarding MMJ?” (Braun et al. 2018).
- “Self-reported competency in MMJ pharmacology” (1-7 Likert scale; responses of 5-7 [good, very good, and excellent, respectively] were approved and consolidated for analyses reporting “confidence in knowledge of medicinal cannabis”) (Hwang et al. 2016).
- "Do you feel confident regarding your current knowledge of [cannabinoids]? (responses of “confident” and “somewhat confident” were approved and consolidated for analyses reporting “confidence in knowledge of medicinal cannabis”) (Ablin et al. 2016; Fitzcharles et al. 2014).
- “Knowledge of pharmacology and indications" (responses indicating a “medium-high” or “high” level of knowledge were approved and consolidated for analyses reporting “confidence in knowledge of medicinal cannabis”) (Ebert et al. 2015).
- “How would you rate your knowledge on the systemic effects of cannabis?” (Crosby 2018).
- “Rate your knowledge on factual information regarding marijuana" (responses indicating “moderate” and “high” levels of knowledge were approved and consolidated for analyses reporting “confidence in knowledge of medicinal cannabis”) (Burke and Marx 1971).

- Research question 6: Do you desire additional education regarding MMJ and/or do believe that education on (medical) cannabis should be made readily available to medical professionals?

Qualified survey questions included the following phrases:

- “(More) training about medical marijuana should be incorporated into medical/pharmacy school curriculum” (Bega et al. 2016; Caligiuri et al. 2018; Chan et al. 2017; Moeller and Woods 2015).
- “Continuing medical education (CME) about medical marijuana should be made available to (primary care) physicians” (Carlini et al. 2016; Ebert et al. 2015; Kondrad and Reid 2013).
- “People in my position should receive education about cannabis” (1-5 Likert; responses of “somewhat agree” and “fully agree” were approved and consolidated for analyses reporting “yes” for the stated research question) (Norberg et al. 2012).
- “Do you feel that more education about marijuana is needed?” (Szyliowicz and Hilsenrath 2019).
- “Are you interested in learning more about MC?” (Philpot et al. 2019; Zylla et al. 2018).
- “It would be helpful to have additional education about MMJ” (Rapp et al. 2015).
- “How strong is the need for education on CTP?” (responses reporting a “strong” or “very strong” need were approved and consolidated for analyses reporting “yes” for the stated research question) (Balneaves et al. 2018; Ziemianski et al. 2015).
- “Dispensing cannabis in the pharmacy requires additional education” (Stojanovic et al. 2017).

- Research question 7: Are you concerned about cannabis’ dependence/addiction potential?

Qualified survey questions included the following phrases:

- “(Do you believe that) marijuana can be addictive (yes/no)?” (Carlini et al. 2016; Chan et al. 2017; Kondrad and Reid 2013; Kusturica et al. 2019; Uritsky et al. 2011).
- I am concerned with MC’s potential for addiction or its psychoactive problems (those reporting “moderate” or “high” concern were approved and consolidated for analyses reporting concern) (Ricco et al. 2017).
- “Are you concerned about substance abuse among patients who receive MMJ?” (Ananth et al. 2018).
- “Are you concerned with MMJ’s potential for abuse/misuse/diversion?” (Rapp et al. 2015).
- “Do you believe / are you concerned that addiction and dependence are potential side effects of MC?” (Karanges et al. 2018; Martins-Welch et al. 2017; Stojanovic et al. 2017).
- “On the scale of 1-7 (1 = no concern, 7 = most concern), how concerned are you about the psychoactive effect and potential addiction from cannabis use?” (responses of 5-7 were approved and consolidated for analyses reporting “concern about MMJ’s addictive potential”) (Hwang et al. 2016).
- “The risk of addiction/physiological dependence would reduce my willingness to prescribe MMJ (1-5 Likert scale [1 = would not reduce my prescribing, 5 = would greatly reduce my prescribing]; responses of 4 and 5 were approved and consolidated for analyses reporting “concern about MMJ’s addictive potential”) (Jacobs et al. 2018).

## Abbreviation

MC: Medical/medicinal cannabis
